# SspE-mediated immune defense: GTP hydrolysis as an allosteric switch coupling phosphorothioate recognition to DNA cleavage

**DOI:** 10.1128/mbio.00359-26

**Published:** 2026-05-12

**Authors:** Yufeng Zhou, Kuo Zhang, Yu He, Haiyan Gao, Yuhang Zhong, Xiaoguang Wang, Meiying Wang, Lianrong Wang, Shi Chen

**Affiliations:** 1 Department of Gastroenterology, Ministry of Education Key Laboratory of Combinatorial Biosynthesis and Drug Discovery, Hubei Clinical Center and Key Laboratory of Intestinal and Colorectal Disease, Zhongnan Hospital of Wuhan University, School of Pharmaceutical Sciences, Wuhan University 271677 , Wuhan, Hubei, China; 2 Department of Respiratory Diseases, Institute of Pediatrics, Shenzhen University Medical School, Shenzhen Children's Hospital, Shenzhen, China; 3 Department of Surgery, Affiliated Hospital of Jiaxing University 417382, Jiaxing, Zhejiang, China; 4 Department of Rheumatology and Immunology, Shenzhen Key Laboratory of Microbiology in Genomic Modification & Editing and Application, Shenzhen Institute of Translational Medicine, Shenzhen University Medical School, The First Affiliated Hospital of Shenzhen University 481870, Shenzhen, China; Massachusetts Institute of Technology, Cambridge, Massachusetts, USA

**Keywords:** DNA phosphorothioation, anti-phage defense, allosteric regulation

## Abstract

DNA phosphorothioate (PT) modification is an epigenetic mark that enables bacteria to discriminate self from non-self DNA, directing restriction effectors to cleave unmodified foreign DNA. In the PT-dependent Ssp system, SspE acts as the restriction effector that recognizes PT marks to block phage propagation. While the mechanism of the Streptomyces homolog (StSspE) is known, the basis for the exceptional potency of *Escherichia coli* (*E. coli*) 3234/A SspE (EcSspE) remained unclear. Here, we combine cryo-electron microscopy (cryo-EM), biochemistry, and functional assays *in vivo* to define its mechanism. The cryo-EM structure reveals that EcSspE forms a dynamic homotetramer with a side-by-side assembly, featuring a substantially reduced inter-subunit interface compared to the intertwined StSspE tetramer. A hydrophobic cavity harboring Y63 specifically recognizes the 5′-C_PS_CA-3′ PT motif. This recognition triggers GTP hydrolysis via the essential residue R133. Hydrolysis, in turn, drives an asymmetric allosteric rearrangement that licenses the flexible C-terminal HNH nuclease domain for DNA cleavage. Disrupting PT sensing (Y63A), GTP hydrolysis (R133A), or nuclease activity (N724A) completely abolishes anti-phage defense, confirming strict functional coupling. Our work establishes a conserved “recognize–hydrolyze–activate” paradigm for SspE proteins, wherein PT-stimulated GTPase activity licenses the nuclease via an allosteric switch. The distinct tetrameric architecture of EcSspE likely underlies its enhanced activity by facilitating conformational dynamics. This study elucidates the precise molecular logic of a potent bacterial immune system and provides a framework for engineering phage resistance.

**IMPORTANCE:**

Bacterial antiphage defense systems must precisely destroy invaders while avoiding self-harm. This study provides a high-resolution molecular blueprint of the exceptionally potent PT-dependent Ssp system from *E. coli* 3234/A. We elucidate its conserved “recognize–hydrolyze–activate” mechanism: the effector EcSspE integrates PT recognition, GTP hydrolysis, and allosteric signaling to license DNA cleavage. Beyond this paradigm, we reveal that subtle evolutionary refinements in its quaternary architecture—a streamlined, side-by-side assembly with a reduced interface—amplify defensive output by enhancing conformational dynamics. This insight bridges structural biophysics and immunity. The system’s strict PT-dependence ensures biosafety, and its defined mechanistic logic and key molecular switches (Y63, R133, N724) establish a framework for engineering programmable phage resistance, advancing both our understanding of host-virus conflict and our ability to harness it.

## INTRODUCTION

The ongoing evolutionary arms race between bacteria and bacteriophages has driven the emergence of diverse bacterial defense strategies, including restriction-modification (1), CRISPR-Cas systems (2, 3), and abortive infection (Abi) (4, 5). Recent advances in bioinformatics, metagenomics, and cryo-electron microscopy (cryo-EM) have dramatically expanded the known diversity of bacterial defense mechanisms. Notable examples include the discovery of cyclic oligonucleotide-based antiphage signaling systems (CBASS) (6, 7), Thoeris (8, 9), Gabija (10, 11), Hachiman (12), Hailong (13), Kongming systems (14), and others. Among these, DNA phosphorothioate (PT)-based defense systems—an epigenetic form of immunity involving backbone modification—have garnered significant interest (15, 16). These systems operate through the enzymatic replacement of a non-bridging oxygen atom with sulfur in the DNA sugar-phosphate backbone, creating a stable PT modification (17, 18). This mark not only modulates key physiological processes but also enables the discrimination between self and non-self DNA (16, 19), thereby establishing a robust layer of antiphage immunity (20–23). Conversely, phages counteract these defenses either by inactivating host defense proteins or by evading them through genome modification (24, 25).

Among these PT-based systems, the Dnd and Ssp systems employ fundamentally distinct defense mechanisms. The Dnd system installs double-stranded PT modifications, and its effector complexes (e.g., *DndFGH*) restrict unmodified “non-self” DNA in an ATP hydrolysis-dependent manner (21). In contrast, the Ssp system catalyzes single-stranded PT modification and operates via a direct “sense–hydrolyze–activate” mechanism (26). This paradigm is exemplified by the canonical system from *Vibrio cyclitrophicus* FF75, where the *sspABCD* complex incorporates PT at 5′-C_PS_CA-3′ motifs (PS, phosphate-sulfur linkage) with a frequency (~14%) higher than that of Dnd at its cognate sites (~12%) (27). Central to this system is the core effector SspE, a dual-function protein whose N-terminal GTPase activity is specifically stimulated by PT-DNA (“self” signal), thereby licensing its C-terminal HNH nuclease to cleave unmarked foreign DNA (26). Critically, unlike traditional restriction endonucleases or Dnd effectors, SspE exhibits no toxicity in the absence of host PT modifications, highlighting its stringent dependence on the “self” mark and exceptional biosafety (28).

Despite this shared safety principle, the protective strength of the Ssp system varies markedly among bacteria. Our previous work demonstrated that the *sspBCDE* system from *E. coli* 3234/A—encoded by a streamlined gene cluster—could restrict phage propagation by up to 10⁶-fold when engineered into industrial strains, indicating exceptionally broad and potent activity (29). This striking potency stands in marked contrast to the significantly weaker protection observed with Ssp systems from *V. cyclitrophicus* FF75, which retains the canonical *sspABCDE* cluster, and from *Streptomyces yokosukanensis* DSM 40224 (20, 26). Notably, in the *E. coli* 3234/A strain used in this study, the *ssp* defense locus is restricted to the *sspBCDE* gene cluster and lacks an encoded *sspA* gene. The requisite cysteine desulfurase activity is instead supplied by the host-encoded IscS enzyme, a genomic ortholog that fulfills the functional role of SspA (28). This genetic configuration highlights the modularity and functional independence of the core *sspBCDE* effector module. Although structural and functional studies of StSspE have revealed its PT-DNA-activated dual-function architecture, the mechanism by which the highly potent EcSspE coordinates these activities remains elusive (26).

To address this gap, we implemented an integrated strategy combining high-resolution cryo-EM, biochemistry, and *in vivo* assays. We aimed to investigate the evolutionary conservation of SspE’s mechanism and to decipher its allosteric code. The system couples epigenetic PT mark recognition to effective DNA cleavage via a GTP-dependent conformational switch, ultimately executing anti-phage defense. Our work represents a paradigm shift by elucidating the conserved logic of SspE-mediated immunity, which is both robust and tightly regulated. This discovery provides a distinct conceptual framework for understanding bacterial defense mechanisms and a fresh perspective on their evolution.

## RESULTS

### Cryo-EM structure reveals the tetrameric assembly of EcSspE

**Fig 1:**
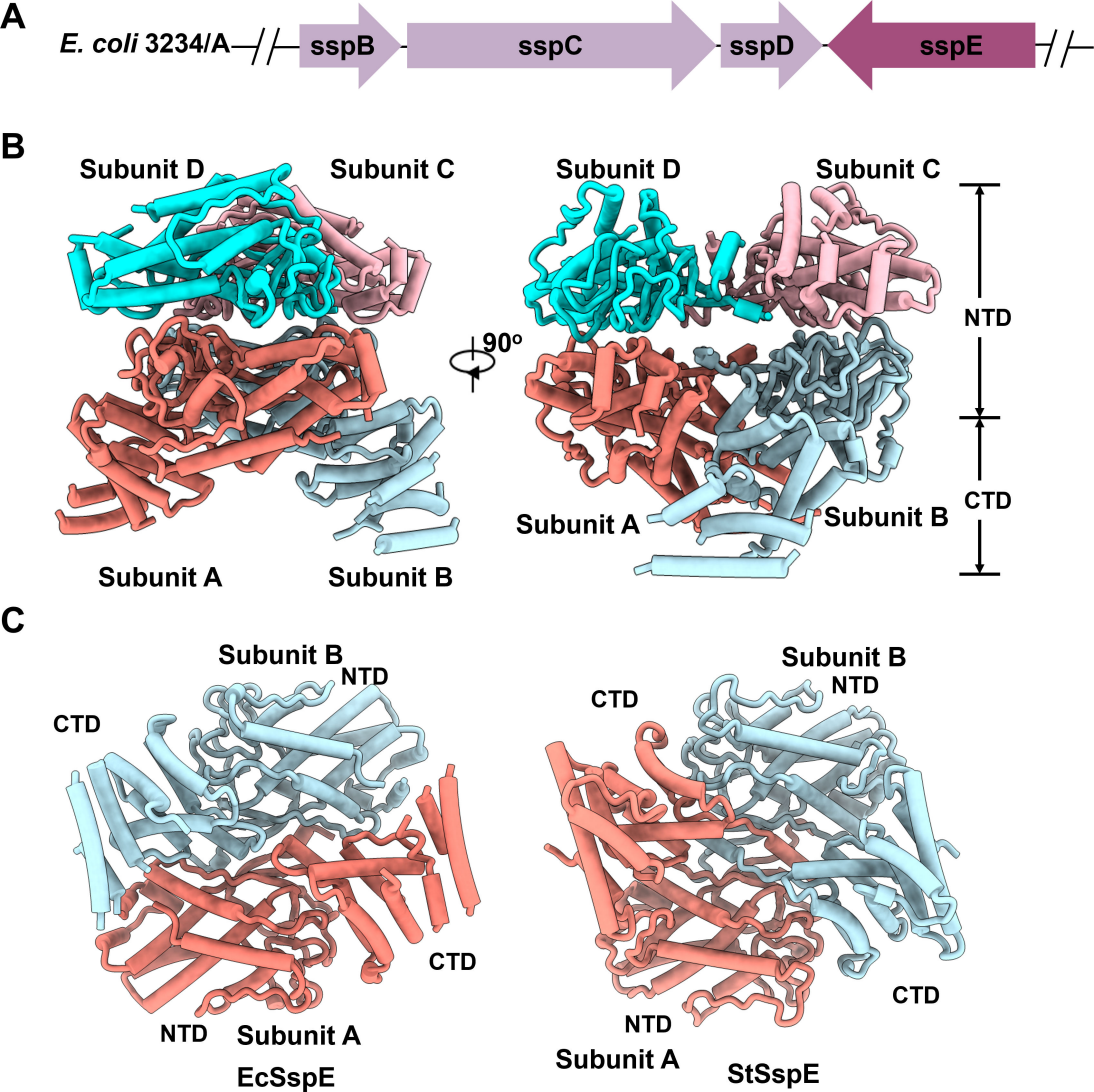
Tetrameric architecture of EcSspE and dimer interface comparison with StSspE. (**A**) Genomic locus of the *sspBCDE* defense system in *E. coli* 3234/A. (**B**) Cryo-EM structure of the EcSspE homotetramer. Subunits are colored: A, deep salmon; B, light blue; C, light pink; D, pale cyan. (**C**) Subunit assembly architectures of EcSspE (left) and StEcSspE (right).

To elucidate the molecular mechanism of SspE, the effector protein encoded within the *sspBCDE* defense locus (Fig. 1A), we determined the cryo-EM structure of *E. coli* 3234/A SspE (EcSspE, 818 aa) at 3.28 Å resolution (Fig. 1B; Fig. S1; Table S1). The resulting structural model reveals that EcSspE assembles into a tetramer (Fig. 1B), a quaternary architecture that is conserved in its *Streptomyces* homolog (StSspE, 771 aa) despite their low sequence identity of only 40%. The EcSspE tetramer displays significant conformational heterogeneity. In the cryo-EM structure, while subunits A and B are well-resolved for residues 34–576, only the N-terminal regions (residues 34–365) are visible in subunits C and D (Fig. 1B). The remaining C-terminal segments lack interpretable electron density, indicating considerable structural flexibility. The remaining C-terminal segments lack interpretable electron density, indicating considerable structural flexibility. This flexibility appears to be a conserved feature of SspE proteins, as a similar absence of well-defined electron density has been reported for the C-terminal region (residues 327–771) of the *Streptomyces* homolog StSspE (20, 26). The observed flexibility is mechanistically consistent with the functional role of the HNH domain as a tightly regulated module that must undergo a large conformational change upon activation. This consistency suggests that inherent structural dynamics, which may involve conformational changes during the functional cycle, are intrinsic to SspE proteins and prevent the visualization of a fully ordered state.

Although EcSspE and StSspE share high overall structural similarity (RMSD = 2.17 Å) and conserved sequence motifs (Fig. S3), they adopt markedly different assembly architectures. EcSspE adopts a relatively symmetric, side-by-side arrangement with its N- and C-terminal domains (NTDs and CTDs) positioned on the same side. In contrast, StSspE assumes a partially intertwined configuration characterized by interwoven NTDs (Fig. 1C). Structural superposition reveals that this divergence originates from the opposing behavior of the flexible inter-domain linker during assembly: it extends clockwise from the NTD in StSspE but folds back counterclockwise in EcSspE (Fig. S2). Consequently, EcSspE has a substantially reduced inter-subunit interface (~1,700 Å² vs ~2,600 Å² in StSspE), potentially facilitating the conformational mobility required for PT sensing and DNA cleavage. These distinct interfacial features suggest differences in assembly stability and allosteric regulation between the two homologs.

### GTPase and nickase activities of EcSspE

**Fig 2:**
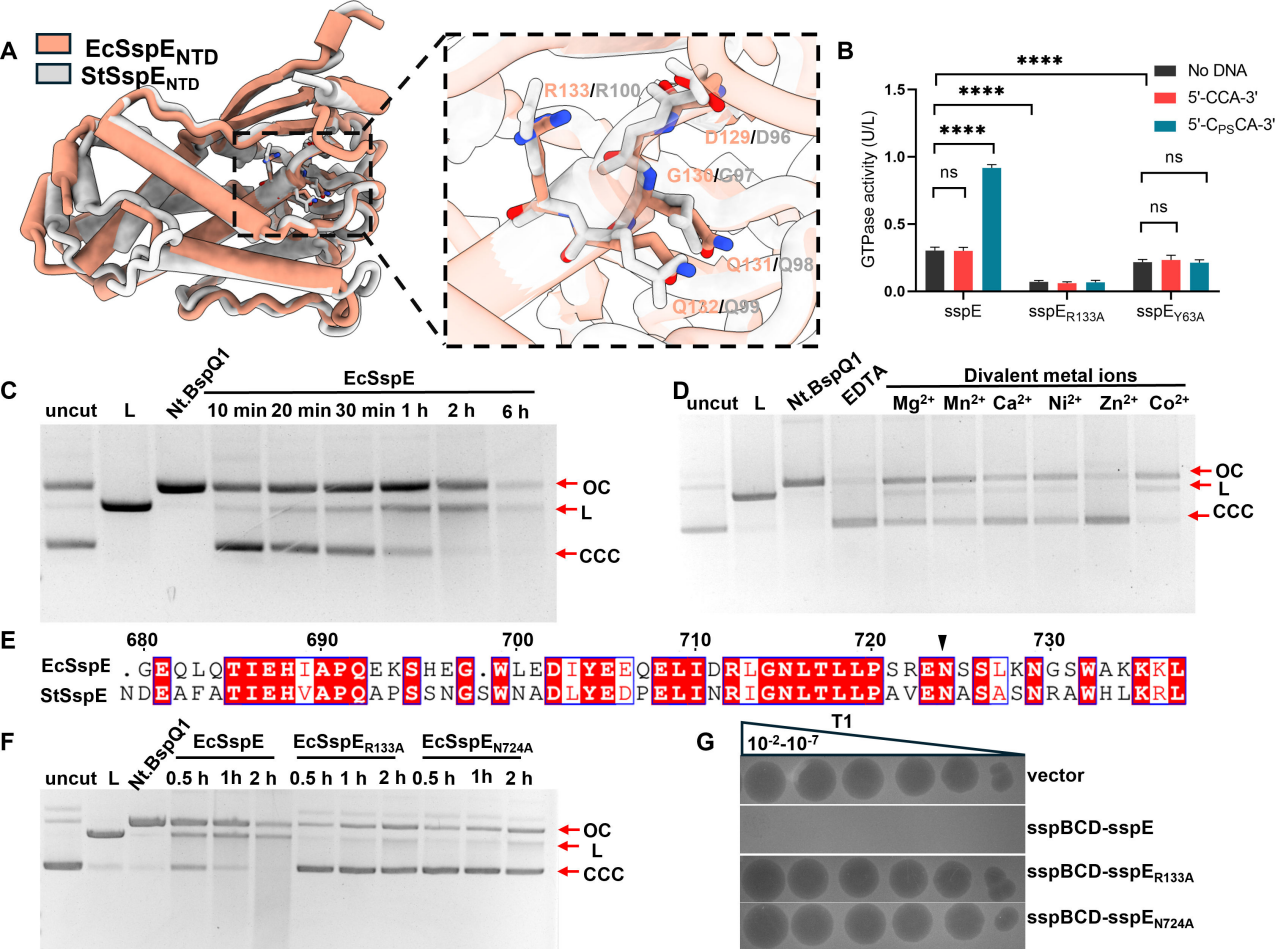
GTPase and DNA nickase activities of EcSspE. (**A**) Structural comparison of the NTDs from EcSspE and StSspE. The close-up view (inset) highlights the conserved DGQQR motif, with EcSspE shown in light red and StSspE in light gray. (**B**) GTPase activity of wild-type EcSspE and its mutants was assessed in the presence of 40-bp DNA fragments containing either an unmodified 5′-CCA-3′ sequence or a PT-modified 5′-C_PS_CA-3′ sequence. Data are presented as mean ± SEM from three independent experiments. Statistical significance was determined by unpaired, two-sided Student’s tests ****P < 0.0001, NS, not significant. (**C**) DNA nicking activity of EcSspE. pUC19 plasmid (300 ng) was incubated with 2 μM EcSspE for the indicated times (10 min to 6 h). Controls: Nt.BspQI-nicked (OC) and BamHI-linearized (L) plasmids. CCC, covalently closed circular. (**D**) Divalent cation dependence of EcSspE nicking activity. (**E**) Sequence alignment of the C-terminal HNH nuclease domains from EcSspE (*E. coli* 3234/A) and StSspE (*S. yokosukanensis* DSM 40224). (**F**) Nicking activity assays for the wild-type and EcSspE variants R133A, N724A on pUC19 DNA. (**G**) Plaque assays were performed to assess the anti-phage activity of *E. coli* MG1655 strains expressing *sspBCD* with either wild-type *sspE* or its mutants. Bacteria were challenged with 1.5 μL of serial 10-fold dilutions (10^−2^ to 10^−7^) of phage T1 and incubated at 37°C.

To delineate the functional coupling between GTP hydrolysis and nuclease activation in EcSspE, we characterized the enzymatic activities of key point mutants (26). Structural analysis revealed that the NTDs of both EcSspE and StSspE adopt the characteristic DUF262 fold, which is part of the ParB-like superfamily encompassing proteins involved in diverse biological processes, and contain a strongly conserved DGQQR signature motif (Fig. 2A) (20, 23, 26, 30–32). A close-up view highlighted the high degree of preservation of this motif (Fig. 2A, right panel), suggesting its critical role in GTP hydrolysis. To functionally characterize this activity and its regulation, we measured the GTPase activity of wild-type and mutant proteins. The results showed that wild-type EcSspE exhibits basal GTP hydrolysis, which was stimulated approximately threefold by the addition of PT-modified DNA containing the 5′-C_PS_CA-3′ motif. Furthermore, the conserved DGQQR motif proved essential for GTP hydrolysis in both homologs. Consistent with this functional conservation—and mirroring the loss of activity in the *Streptomyces* homolog (R100A)—the equivalent R133A mutation in EcSspE also completely abolished GTP hydrolysis (Fig. 2B). These findings underscore the importance of the conserved DGQQR motif and establish that the positive charge at this arginine residue is essential for the GTP hydrolysis activity of SspE across species.

We also characterized the nuclease function and enzymatic properties of the C-terminal HNH domain in EcSspE. Time-course assays showed that EcSspE efficiently converts supercoiled pUC19 plasmid into the open-circular form within 1 h (Fig. 2C), indicating rapid and strong intrinsic DNA nicking activity. This activity was strictly dependent on specific divalent metal ions (e.g., Mg^2+^, Mn^2+^, Co^2+^), with no cleavage detected in the presence of Zn^2+^ (Fig. 2D), consistent with the need for a potent, metal-dependent nuclease in rapid anti-phage defense. Notably, the R133A mutation simultaneously abolished both GTP hydrolysis and DNA nickase activities (Fig. 2B and F), indicating that this residue is indispensable for coordinating the dual enzymatic activities of the protein. Furthermore, sequence alignment identified N724 in EcSspE as the equivalent catalytic residue to N676 in StSspE (Fig. 2E). Consistent with this functional conservation, the N724A mutation also resulted in a complete loss of DNA nicking activity (Fig. 2F). Most importantly, phage plaque assays demonstrated that both the R133A and N724A mutants completely lost the ability to restrict phage propagation (Fig. 2G), confirming that the catalytic integrity of both the GTPase and nuclease domains is essential for anti-phage defense *in vivo*. This functional impairment at the cellular level is fully consistent with our biochemical observations that these mutations abolish their respective enzymatic activities. Collectively, these findings establish that EcSspE possesses a potent, metal-dependent nuclease activity that is functionally coupled to its GTPase domain through R133, while maintaining evolutionary conservation of its catalytic core via N724, with all of these elements being indispensable for robust anti-phage immunity.

### The R133A mutation reveals an asymmetric conformational mechanism in EcSspE for cooperative DNA engagement

**Fig 3:**
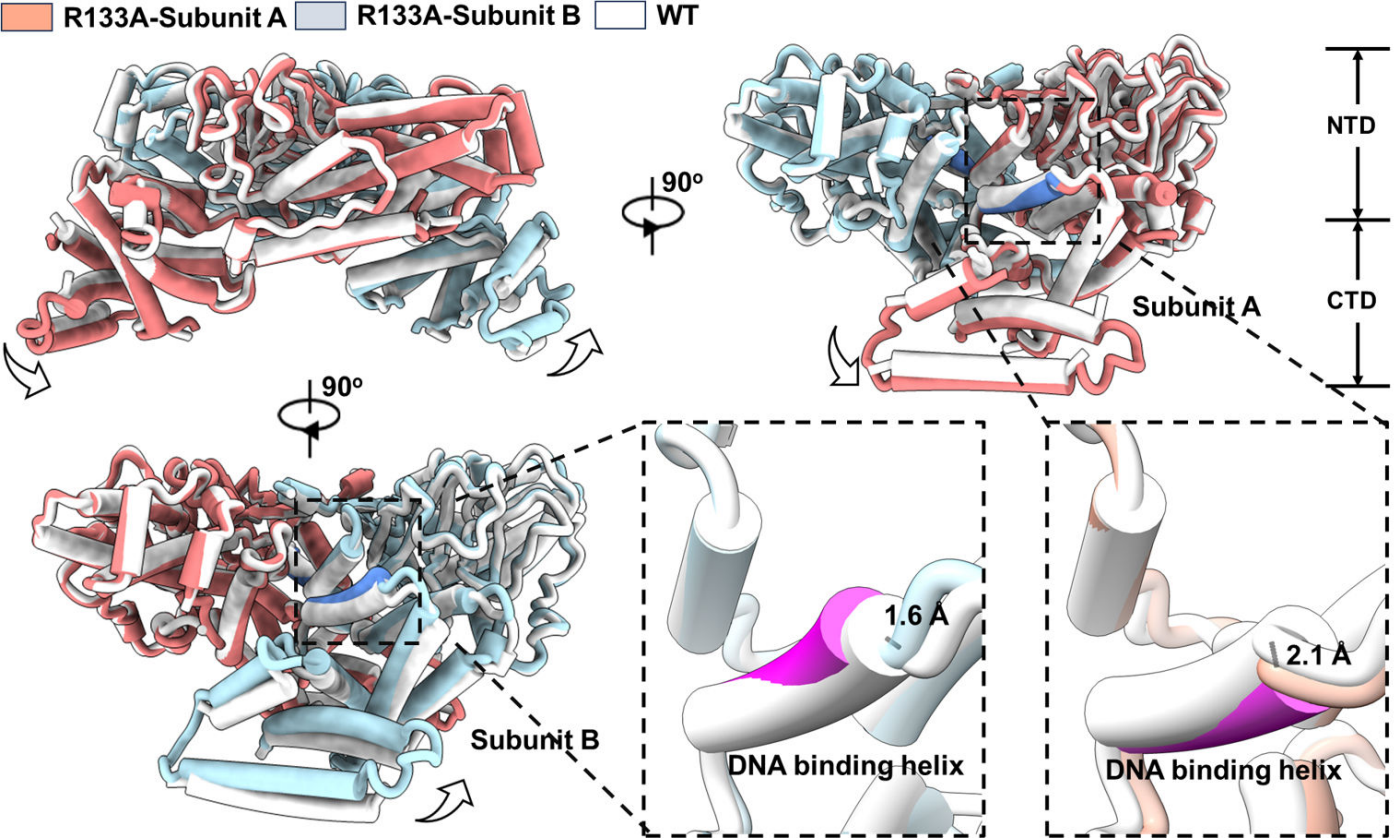
Structure comparison of the EcSspE_R133A_ with EcSspE_WT_. Subunit A and B of EcSspE_R133A_ and EcSspE_WT_ are colored in deep salmon, light blue, and white, respectively. The DNA binding helix is highlighted in magenta.

GTP hydrolysis causes StSspE to dissociate from PT-DNA, while inhibiting GTPase hydrolytic activity (R100A) induces an allosteric change in StSspE, enhancing its binding to substrate DNA (26). To investigate whether EcSspE employs an allosteric regulatory mechanism similar to StSspE, we determined the cryo-EM structure of the GTPase-critical mutant EcSspE_R133A_ at an overall resolution of 2.81 Å. Notably, the C-terminal domain (residues ~650–791) encompassing the HNH nuclease module also lacked defined density in the EcSspE_R133A_ structure, confirming that this flexibility is a reproducible feature across different functional states (Fig. 3; Fig. S1; Table S1). Together with the conserved disorder in StSspE, this indicates that inherent dynamics are an intrinsic property of the SspE family. This observed flexibility is mechanistically consistent with the functional role of the HNH domain as a tightly regulated module that must undergo a large conformational change upon activation.

The structural superposition of the NTDs of the wild-type and R133A mutant revealed that inhibiting GTPase hydrolytic activity induces asymmetric conformational changes in the CTDs of both subunits A and B (Fig. 3). Relative to the stable NTDs, the CTDs of both subunits A and B undergo a slight counterclockwise rotation. Concurrently, the DNA-binding helix on the CTD of subunit A shifts away from its NTD by approximately 2.1 Å, likely to optimize DNA binding, while the corresponding DNA-binding helix on subunit B moves closer to its NTD by about 1.6 Å. This suggests that the two subunits adopt complementary and asymmetric conformations to bind DNA in a coordinated manner. Such a conformational division of labor indicates that they do not act independently but likely engage with the DNA substrate cooperatively through an allosteric cooperative mechanism.

### Structural and functional identification of the hydrophobic cavity harboring the PT-recognizing residue Y63 in EcSspE

**Fig 4:**
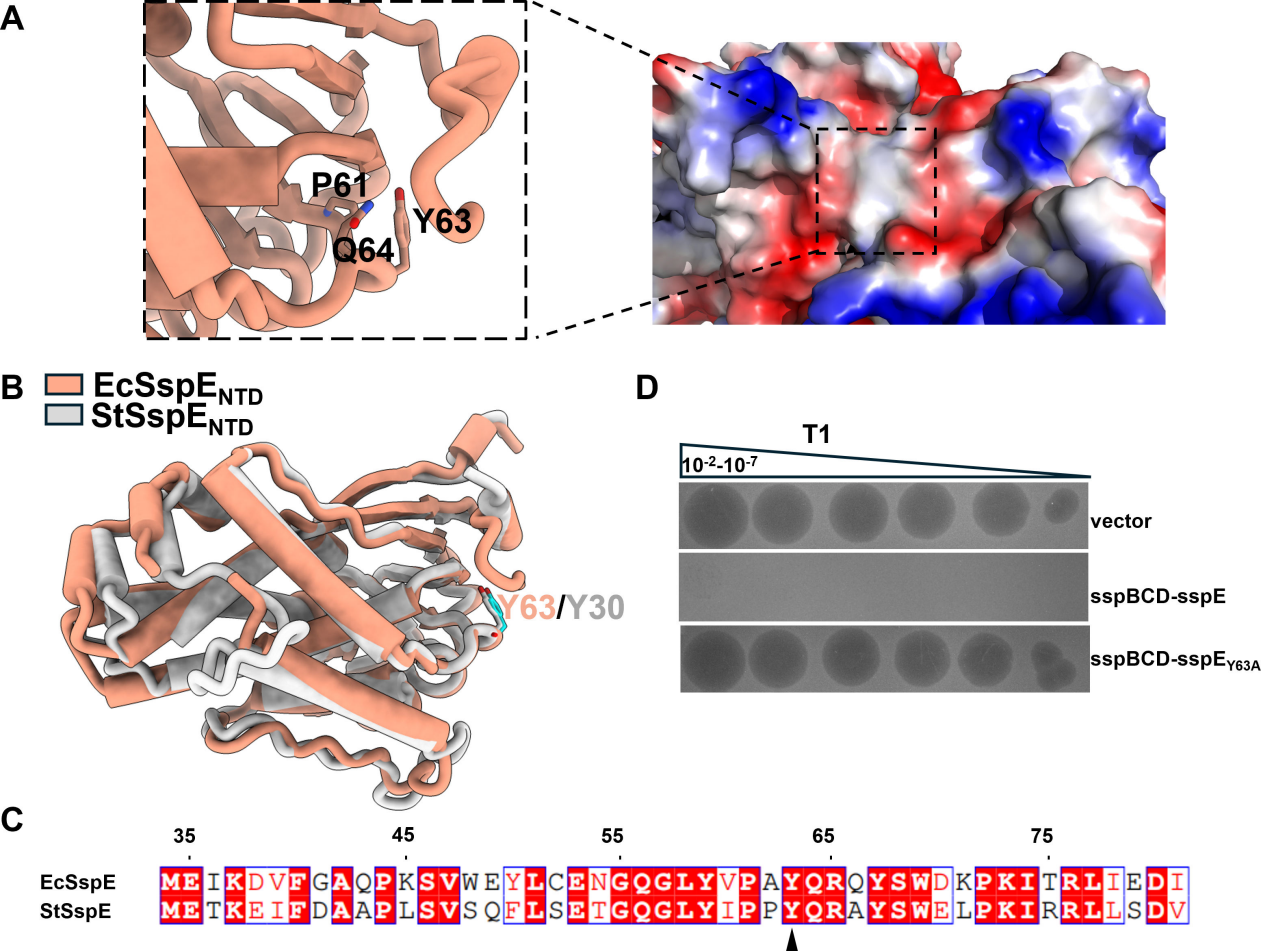
The hydrophobic cavity harboring Y63 for PT recognizing. (**A**) Close-up view of the PT-sensing hydrophobic patch in EcSspE, highlighting key residues P61, Y63, and Q64 (shown as sticks). (**B**) Structural alignment of the N-terminal domains (NTDs) of EcSspE (light red, PDB: 20YV) and StSspE (light gray, PDB: 7DRS). The conserved tyrosine residues Y63 (EcSspE) and Y30 (StSspE) are highlighted in stick representation. (**C**) Multiple sequence alignment of the Y63-flanking regions from EcSspE (WP_000402995.1) and StSspE (WP_067135523.1), demonstrating the conservation (Y63 in EcSspE) of the key tyrosine residue (indicated by a black arrow). (**D**) Plaque assays were performed to assess the anti-phage activity of MG1655 strain expressing SspBCDE_Y63A_. Bacteria were challenged with 1.5 μL of serial 10-fold dilutions (10^−2^ to 10^−7^) of phage T1 and incubated at 37°C.

Structural analysis revealed a well-defined hydrophobic cavity within the EcSspE N-terminal domain (NTD), harboring residues P61, Y63, and Q64 (Fig. 4A). This cavity is structurally analogous to the PT-recognizing patch previously characterized in the StSspE homolog. Furthermore, superposition of the EcSspE and StSspE NTDs further demonstrated a conserved spatial arrangement, particularly for the aromatic ring of the key tyrosine residue (Y63 in EcSspE; Y30 in StSspE) (Fig. 4B). This high degree of structural conservation was supported by multiple sequence alignment, which confirmed the strict conservation of this tyrosine residue across homologs (Fig. 4C). To validate the functional role of Y63 in PT recognition and anti-phage defense, we characterized the GTPase activity of the EcSspE_Y63A_ mutant. The mutant retained basal GTP hydrolysis activity; however, the addition of PT-modified DNA failed to stimulate this activity, indicating a specific loss of the allosteric activation normally triggered by PT recognition (Fig. 2B). We next engineered a bacterial strain harboring the SspBCDE_Y63A_ system. Phage plaque assays demonstrated that, in contrast to the wild-type system, the mutant completely lost the ability to restrict phage T1 propagation (Fig. 4D). Our structural analysis identified a key hydrophobic pocket in EcSspE (Fig. 4A). The functional inactivation of the Y63A mutant (Fig. 2B and 4G) definitively establishes that Y63 within this pocket is essential for recognizing PT modification and initiating the activation cascade.

**Fig 5:**
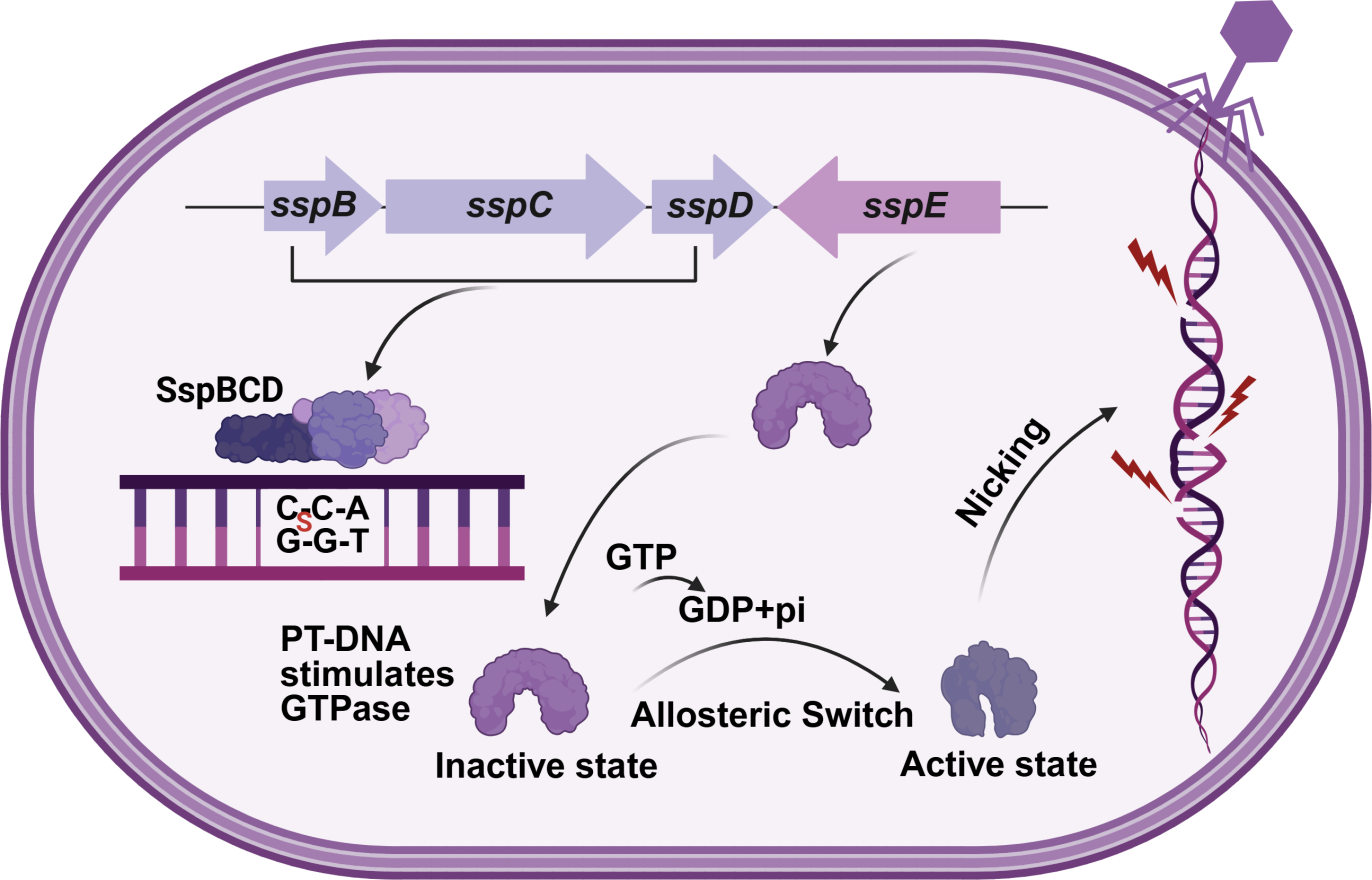
Schematic model of PT-DNA-triggered allosteric activation and anti-phage defense by EcSspE. The SspBCD complex installs phosphorothioate (PT) modifications at specific 5′-CCA/GGT-3′ sites in host DNA. EcSspE, which acts as the restriction effector, remains in an inactive state in the absence of PT-DNA. Binding to PT-modified DNA stimulates its GTPase activity, triggering an allosteric switch to the active state. This conformational switch enables the HNH nuclease domain to cleave invasive, non-PT-modified phage DNA, thereby providing defense while avoiding host-genome damage.

In summary, our findings establish a PT-dependent defense mechanism whereby EcSspE licenses its HNH nuclease to cleave invasive DNA. Recognition of host PT modifications stimulates EcSspE’s GTPase, triggering an allosteric switch from an inactive to an active state, thereby ensuring immunity while preventing self-targeting (Fig. 5).

## DISCUSSION

Our study elucidates the molecular mechanism of EcSspE, a key effector in a potent anti-phage defense system. We demonstrate that EcSspE confers immunity through strict dependence on host DNA phosphorothioate (PT) modification, ensuring exceptional biosafety. High-resolution cryo-EM reveals its tetrameric architecture, which features a side-by-side assembly with a reduced inter-subunit interface compared to the partially intertwined arrangement of StSspE. We propose that this distinct architecture may grant EcSspE greater conformational mobility, thereby facilitating its superior activity. This hypothesis is supported by the observed high intrinsic flexibility of the HNH domain and the asymmetric conformational shifts induced in the GTPase-deficient R133A mutant, both of which are consistent with a more dynamic tetrameric scaffold.

Despite low sequence identity, EcSspE shares a conserved core mechanism with StSspE. A dedicated hydrophobic cavity harboring Y63 specifically recognizes the 5′-C_PS_CA-3′ PT mark. This recognition promotes GTP hydrolysis via R133, initiating a conserved allosteric switch. Notably, the C-terminal HNH nuclease domain exhibits significant conformational flexibility across homologs—a prerequisite for transitioning from an auto-inhibited to an active state. GTPase-deficient mutations induce asymmetric movements in the HNH domains, suggesting subunits adopt complementary roles during DNA engagement. The atomic architecture of EcSspE reveals that the core functional modules (the PT-recognizing cavity, GTPase switch, and HNH nuclease fold) are architecturally conserved, a finding underscored by comprehensive sequence alignment (Fig. S3), providing a definitive physical framework for the SspE family. Mutational analysis confirms that disrupting any key step—PT recognizing (Y63), GTP hydrolysis (R133), or nuclease catalysis (N724)—abolishes defense, underscoring the precision of this sequential activation pathway. Although direct structural evidence for the post-hydrolysis activated state of the HNH domain remains elusive, our integrated data—including strict functional coupling (e.g., R133A, Y63A), asymmetric conformational shifts in the R133A mutant, and the conserved intrinsic flexibility of the HNH domain—support a model in which GTP hydrolysis triggers its large-scale allosteric rearrangement.

Thus, our work supports a model in which the “recognize–hydrolyze–activate” paradigm—originally proposed from biochemical studies—is structurally consolidated within a key model organism. The atomic architecture of EcSspE reveals that the core functional modules (the PT-recognizing cavity, GTPase switch, and HNH nuclease fold) are architecturally conserved, providing a definitive physical framework for the SspE family. Collectively, the reduced interface (~1,700 vs ~2,600 Å² in StSspE), the flexible HNH domain, and the primed conformational state of the R133A mutant support a working model in which EcSspE’s distinct quaternary assembly enhances functional potency by facilitating allosteric dynamics. We note that alternative factors, such as differences in DNA binding or fine-tuning of the allosteric pathway, may also contribute and are not mutually exclusive with this interpretation. Crucially, this structural foundation pinpoints the specific refinements—such as its distinct quaternary assembly—that underlies EcSspE’s superior efficacy, extending the previous model beyond mere conservation to explain enhanced function.

In summary, EcSspE operates as a precisely regulated allosteric machine in which PT-DNA recognizing licenses nuclease activity via GTP-driven conformational switching. The streamlined *E. coli* 3234/A *sspBCDE* system, with its potent stand-alone activity and lack of host toxicity, presents an attractive platform for engineering phage-resistant strains. Its compatibility with other PT systems (e.g., Dnd, *sspFGH*) (33), which can erect sequential PT-modification barriers for synergistic defense, highlights the remarkable modularity of PT-based immunity. The mechanistic logic elucidated here, particularly the roles of key residues like Y63 and R133, offers a blueprint for designing tunable molecular switches and multi-layered defense packages. Future efforts will aim to establish a causal link between interface geometry, dynamics, and function. Direct tests could include (i) engineering the interface (e.g., through residue swaps) to modulate activity; (ii) measuring conformational dynamics via hydrogen-deuterium exchange mass spectrometry (HDX-MS) combined with computational approaches such as molecular dynamics (MD) simulations; and (iii) employing pre-steady-state kinetics to pinpoint the rate-limiting step enhanced in EcSspE. Exploring phage-encoded inhibitors will also be crucial for assessing the system’s evolutionary durability.

## MATERIALS AND METHODS

### Bacterial strains, bacteriophages, and plasmids

All bacterial strains, bacteriophages, and plasmids used in this study are listed in Table S1. *E. coli* DH5α and *E. coli* BL21 (DE3) were used for plasmid construction and protein expression, respectively. *E. coli* MG1655-WT and *E. coli* MG1655 (pWHU3640) were used as hosts for phage-related experiments. The *E. coli* strains were grown in Luria-Bertani (LB) medium (10 g/L tryptone, 5 g/L yeast extract, and 10 g/L NaCl) or on LB agar plates (15 g/L agar) at 37°C. When appropriate, the medium was supplemented with kanamycin (50 µg/mL) and ampicillin (100 µg/mL).

### Plasmid construction

To generate both protein expression constructs and *in vivo* assay vectors, site-directed mutants (Y63A, R133A, and N724A) were constructed using the same sets of mutagenic primers (Table S2). For protein purification, mutations were introduced into the EcSspE gene in plasmid pWHU6001 via overlap-extension PCR. The resulting fragments were ligated into pET28a, yielding plasmids pWHU6002, pWHU6003, and pWHU6004 for expressing His-tagged proteins. For *in vivo* phage infection assays, the corresponding mutations were introduced into the *sspE* gene within the full *sspBCDE* operon using site-directed mutagenesis. The wild-type *sspBCDE* plasmid (e.g., pWHU6004) was used as the template with the identical primer sets. The resulting mutant constructs were designated pWHU6005, pWHU6006, and pWHU6007 and were transformed into *E. coli* MG1655 for bacteriophage infection assays. All plasmids were verified by DNA sequencing.

### Protein expression and purification

The gene encoding wild-type EcSspE and its mutants was subcloned into pET28a vector along with a C-terminal 6×His tag. The resulting plasmid was transformed into *E. coli* BL21 (DE3) competent cells. The overnight culture of transformed cells was diluted 1:100 (vol/vol) in LB medium and incubated at 37°C until the OD_600_ reached 0.8, followed by induction with 0.2 mM isopropyl β-d-1-thiogalactopyranoside (IPTG) for 16–18 h at 16°C. Cells were harvested by centrifugation and resuspended in lysis buffer (25 mM Tris-HCl, pH 8.0, 150 mM NaCl, and 20 mM imidazole) and then lysed using a cell homogenizer (JNBIO). The lysate was centrifuged at 15,000 × *g* for 1.5 h to obtain a clear lysate, and then the supernatant was subjected to Ni^2+^-NTA-affinity chromatography. The eluted protein was loaded onto a Superdex-200 gel filtration column in a solution containing 25 mM Tris-HCl, pH 8.0, 150 mM NaCl, and 2 mM dithiothreitol. The peak fractions were combined and concentrated at 4°C. The concentrated samples were flash-frozen in liquid nitrogen and stored at −80°C for subsequent assays.

### Cryo-EM sample preparation, data collection, and data processing

For the preparation of cryo-EM samples of SspE/SspE_R133A_, 3.0 μL of purified proteins at a concentration of 2.0 mg/mL was applied onto glow-discharged Quantifoil R1.2/1.3 300 mesh copper grids, blotted for 3.0 s without blot force, and plunge-frozen in liquid ethane using Vitrobot Mark IV (Thermo Fisher Scientific). All data collections were performed on a Thermo Fisher Scientific 300 kV TEM Titan Krios equipped with Gatan K3 direct electron detector. Raw movies were collected in super-resolution mode at a magnification of 105,000 and stored in 32-frame gain-normalized stacks. All movies were imported into and processed with cryoSPARC. After motion correction and dose weighting (bin factor of 2) and CTF estimation, micrographs were subjected to particle picking using a template picker. After one round of 2D classification, particles representing well-defined classes were subjected to one round of heterogeneous refinements. The resulting particle set was then processed through homogeneous refinement without imposing symmetry (C1). The map was subsequently improved by non-uniform refinement, also under C1 symmetry. The final map was sharpened using Phenix.auto_sharpen. Details of data collection and processing are provided in Table S3 and Fig. S1.

### Model building and refinement

The AlphaFold-predicted model of SspE was used as the initial model. The model was further improved through cycles of manual corrections in Coot and followed by real-space refinement (with Ramachandran restraints and secondary structure restraints) in Phenix. Refinement statistics of the model are summarized in Table S3. Figures representing the structural features were prepared in ChimeraX.

### DNA nicking assays

The DNA nicking activity was assayed as described (20). Briefly, 300 ng of pUC19 DNA was incubated with 2 µM EcSspE or its variants in a 15 µL reaction containing CutSmart Buffer (New England Biolabs) at 37°C for 30 min. The reaction was stopped by adding 1.5 µL of 10× gel loading dye (Yeasen, Shanghai, China), and the products were analyzed by 1% agarose gel electrophoresis.

### GTPase assays

GTPase activity was measured as previously reported (26). In brief, a reaction mixture containing 0.5 μM EcSspE (or its mutants) and 100 μM GTP in CutSmart Buffer (total volume 40 μL) was incubated at 37°C for 30 min, in the presence or absence of 1 μM DNA containing a PT-modified (5′-C_PS_CA-3′) or unmodified (5′-CCA-3′) motif (Table S2). Phosphate release was then quantified using the Malachite Green Kit (Beyotime Biotechnology): after adding 160 μL water and 70 μL reagent, the absorbance of the phosphomolybdate complex was read at 630 nm following a 30 min color development at room temperature.

### Phage plaque assay

*E. coli* cells from an overnight culture were diluted 1:100 in LB medium and grown to mid-log phase (OD_600_ = 0.6). For the plaque assay, 500 µL of this culture was mixed with 10 mL of LB soft agar (0.75%, wt/vol) and overlaid onto a pre-coated LB agar plate (1.5%, wt/vol). Phage stocks were serially diluted in SM buffer (100 mM NaCl, 8 mM MgSO_4_, 50 mM Tris-HCl, pH 7.5), and 5 µL aliquots of each dilution were spotted onto the solidified agar surface. After overnight incubation at 37°C, phage plaques were imaged using a ChemiDoc XRS+ System (Bio-Rad).

## Data Availability

The atomic coordinates and structure factors for SspE and SspE_R133A_ have been deposited in the Protein Data Bank under the accession numbers 20YV and 20YW. The corresponding EM maps have been deposited in the Electron Microscopy Data Bank under the accession codes 67420 and 67421.

## SUPPLEMENTAL MATERIAL

Fig. S1 mbio.00359-26-s0001.docx Cryo-EM analysis of EcSspE and EcSspER133A.

Fig. S2 mbio.00359-26-s0002.docx Structural comparison of EcSspE and StSspE.

Fig. S3 mbio.00359-26-s0003.docx Multiple sequence alignment of SspE homologs.

Table S1 mbio.00359-26-s0004.docx Strains, phages, and plasmids used in this study.

Table S2 mbio.00359-26-s0005.docx Primers used in this study.

Table S3 mbio.00359-26-s0006.docx Cryo-EM data collection, refinement, and validation statistics.

ASM does not own the copyrights to Supplemental Material that may be linked to, or accessed through, an article. The authors have granted ASM a non-exclusive, world-wide license to publish the Supplemental Material files. Please contact the corresponding author directly for reuse.
