# Supplementary material for: SspE-mediated immune defense: GTP hydrolysis as an allosteric switch coupling phosphorothioate recognition to DNA cleavage
Source: mBio. 2026 May 12;17(6):e00359-26. doi: 10.1128/mbio.00359-26 (PMC13251355; doi:10.1128/mbio.00359-26)
Supplement: Fig. S1 — Cryo-EM analysis of EcSspE and EcSspER133A. [file mbio.00359-26-s0001.docx]

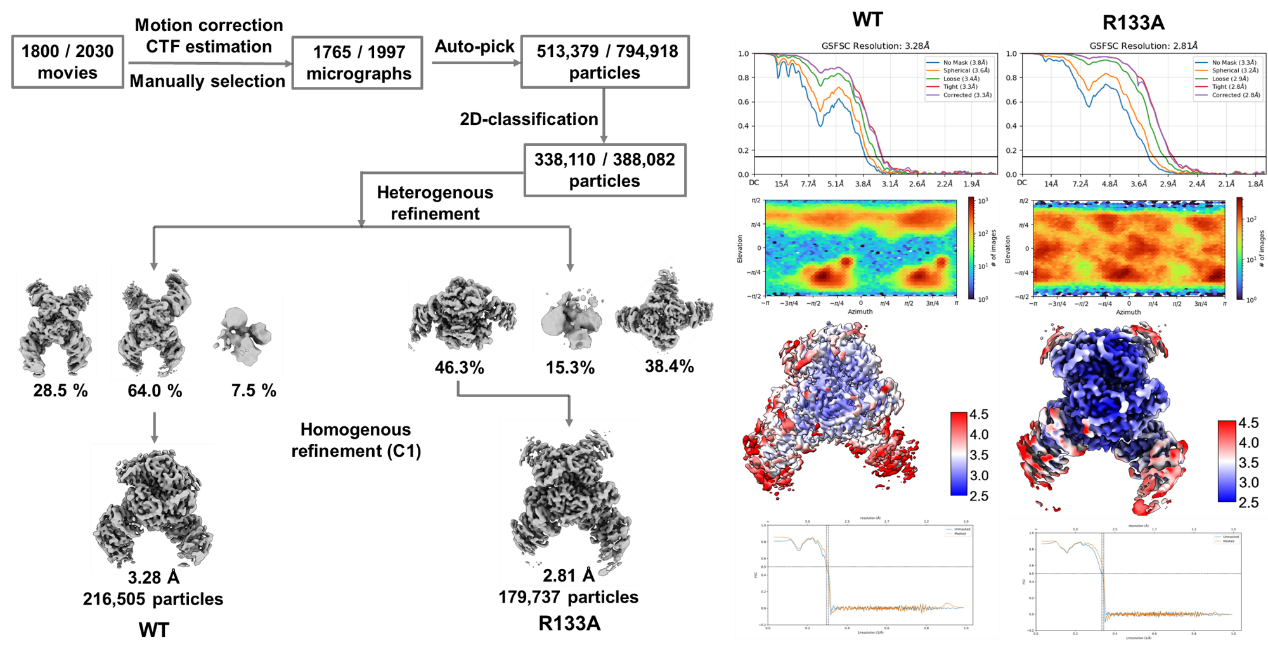


**Fig. S1 Cryo-EM analysis of EcSspE and EcSspE_R133A_.** Left: Flowchart for cryo-EM data processing. Right: FSC curves, the viewing direction distribution plot, local resolution map, and model-to-map FSC of SspE_WT_ and SspE_R133A_.
