# Supplementary figures and images for: SspE-mediated immune defense: GTP hydrolysis as an allosteric switch coupling phosphorothioate recognition to DNA cleavage
Source: mBio. 2026 May 12;17(6):e00359-26. doi: 10.1128/mbio.00359-26 (PMC13251355; doi:10.1128/mbio.00359-26)

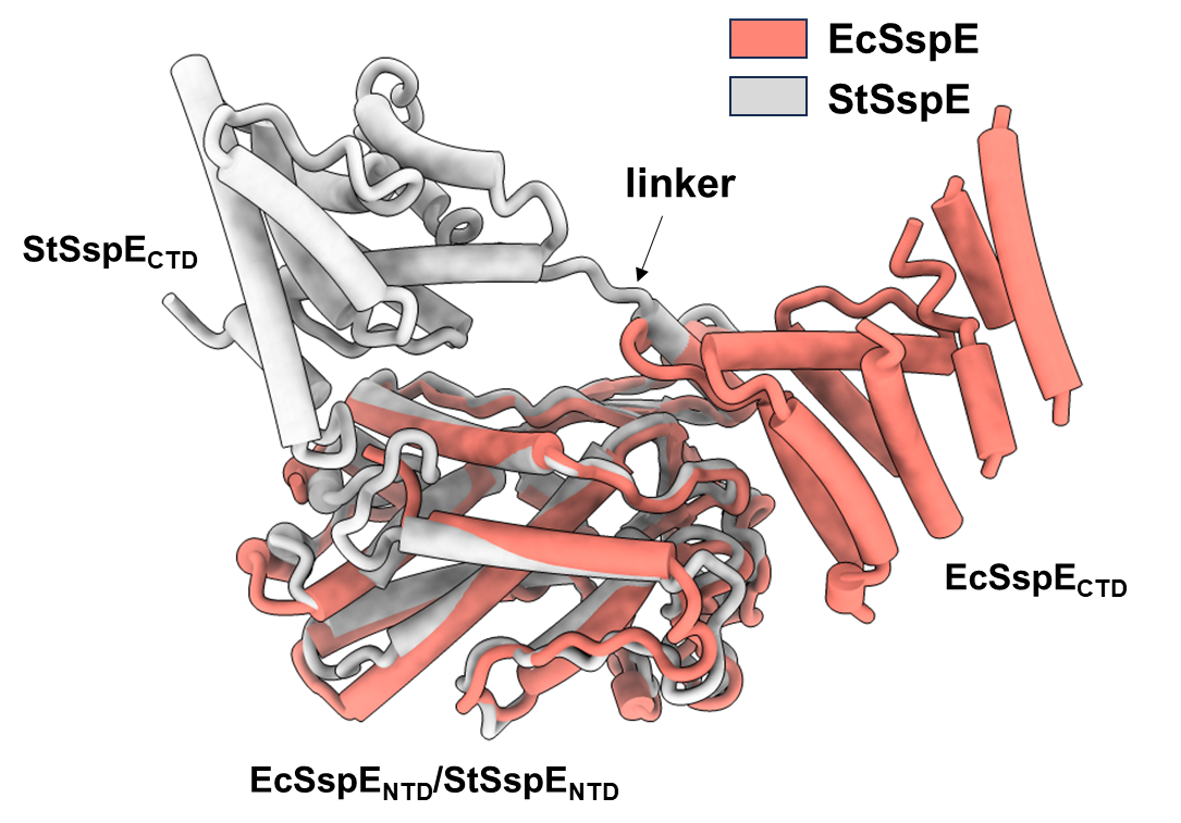


**Fig. S2 Structural comparison of EcSspE and StSspE.**

Supplement: Fig. S2 — Structural comparison of EcSspE and StSspE. [file mbio.00359-26-s0002.docx]
