# Supplementary material for: SspE-mediated immune defense: GTP hydrolysis as an allosteric switch coupling phosphorothioate recognition to DNA cleavage
Source: mBio. 2026 May 12;17(6):e00359-26. doi: 10.1128/mbio.00359-26 (PMC13251355; doi:10.1128/mbio.00359-26)
Supplement: Fig. S3 — Multiple sequence alignment of SspE homologs. [file mbio.00359-26-s0003.docx]

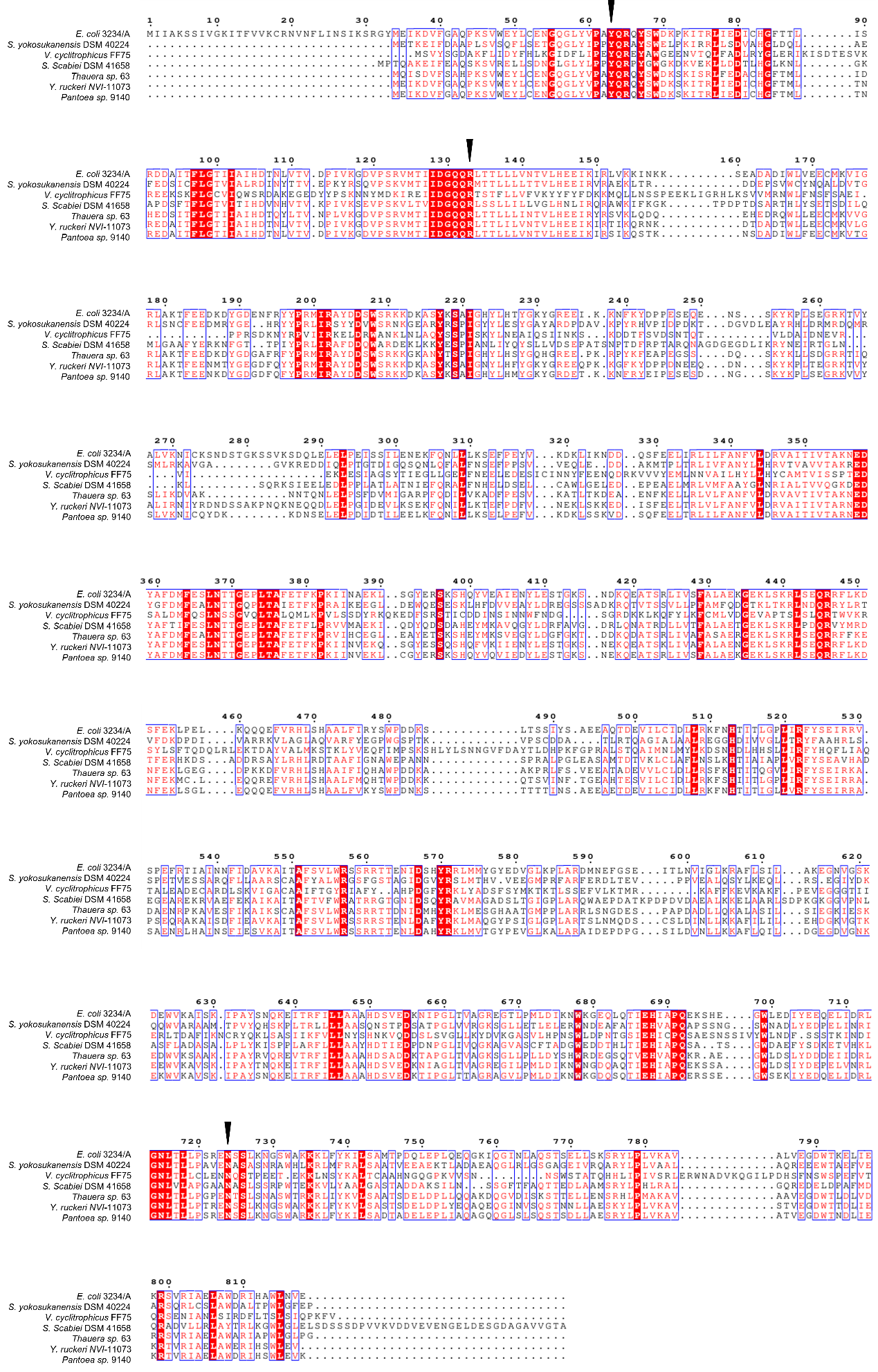


**Fig. S3 Multiple sequence alignment of sspE homologs.**

Full-length alignment of EcSspE, StSspE, and representative homologues reveals strong sequence conservation within key functional domains—including the PT recognizing cavity, GTPase switch, and HNH nuclease fold. Critically conserved residues are marked with black arrows.
