# Supplementary material for: SspE-mediated immune defense: GTP hydrolysis as an allosteric switch coupling phosphorothioate recognition to DNA cleavage
Source: mBio. 2026 May 12;17(6):e00359-26. doi: 10.1128/mbio.00359-26 (PMC13251355; doi:10.1128/mbio.00359-26)
Supplement: Table S1 — Strains, phages, and plasmids used in this study. [file mbio.00359-26-s0004.docx]

**TABLE S1. Strains, phages and plasmids used in this study.**

| **Name** | **Characteristics** | **Source or reference** |
| --- | --- | --- |
| **Strains** |  |  |
| ***E. coli*** |  |  |
| BL21（DE3） | *F- ompT gal dcm lon hsdS_B_(rB-mB-) λ (DE3 [lacIlacUV5-T7p07 ind1 sam7 nin5]) [malB+] K-12(λ^S^) endA1 recA1 gyrA96 thi-1 hsdR17* | Novagen |
| MG1655 | *E. coli K-12, F- λ- rph-1* | PMID:16738553 |
| 3234/A | *d(C_PS_C), GenBank: LCVH01000033* | ATCC |
| DH5α | *F^-^ endA1 glnV44 thi-1 recA1 relA1 gyrA96 deoR nupG purB20 φ80dlacZΔM15 Δ (lacZYA-argF) U169, hsdR17 (rK–mK+),* | Sangon Biotech |
| **Phages** |  |  |
| T1 | *Siphoviridae*,lytic,dsDNA | (1) |
| **Plasmids** |  |  |
| pET28a (±) | expressing vector, His-tag，Km^r^ | Novagen |
| pWHU6001 | pET28a derivative, expressing wild-type SspE from *E. coli* 3234/A | This study |
| pWHU6002 | pET28a derivative, expressing SspE  Y63A from *E. coli* 3234/A | This study |
| pWHU6003 | pET28a derivative, expressing SspE  R133A from *E. coli* 3234/A | This study |
| pWHU6004 | pET28a derivative, expressing SspE  N724A from *E. coli* 3234/A | This study |
| pBluescript II SK (+) | Cloning vector, Amp^r^. | (2) |
| pWHU3640 | pBluescript II SK (+) derivative with an 8 kb HindIII-BamHI fragment carrying sspBCDE from *E. coli* 3234/A, Amp^r^ | (3) |
| pWHU6005 | SK (+) derivative, carrying sspBCDE_Y63A_ from *E. coli* 3234/A, Amp^r^ | This study |
| pWHU6006 | SK (+) derivative, carrying sspBCDE_R133A_ from *E. coli* 3234/A, Amp^r^ | This study |
| pWHU6007 | SK (+) derivative, carrying sspBCDE_N724A_ from *E. coli* 3234/A, Amp^r^ | This study |

**References**

1. Roberts MD, Martin NL, Kropinski AM. 2004. The genome and proteome of coliphage T1. Virology 318:245-66.

2. Alting-Mees MA, Short JM. 1989. pBluescript II: gene mapping vectors. Nucleic Acids Res 17:9494.

3. Zou X, Xiao X, Mo Z, Ge Y, Jiang X, Huang R, Li M, Deng Z, Chen S, Wang L, Lee SY. 2022. Systematic strategies for developing phage resistant Escherichia coli strains. Nat Commun 13:4491.
