# Supplementary material for: SspE-mediated immune defense: GTP hydrolysis as an allosteric switch coupling phosphorothioate recognition to DNA cleavage
Source: mBio. 2026 May 12;17(6):e00359-26. doi: 10.1128/mbio.00359-26 (PMC13251355; doi:10.1128/mbio.00359-26)
Supplement: Table S2 — Primers used in this study. [file mbio.00359-26-s0005.docx]

**TABLE S2. Primers used in this study.**

| **Primers** | **Sequence (5**′**-3**′**)** |
| --- | --- |
| **Construction of pWHU6001** | |
| SspE-1 | TTTAACTTTAAGAAGGAGATATACCATGATTATTGCTAAGTCTTCCATAG |
| SspE-2 | CACCAGGCCGCTGCTCTCGAGTTCAACATTCATTCAACCAAGCATG |
| **Construction of pWHU6002 and pWHU6005** | |
| SspE_Y63A_-1 | GTGCCAGCAGCACAAAGGCAATATTCTTGGGAT |
| SspE_Y63A_-2 | TTGCCTTTGTGCTGCTGGCACATACAATCCTTG |
| **Construction of pWHU6003 and pWHU6006** | |
| SspE_R133A_-1 | GGTCAACAAGCATTGACTACATTGCTATTAGTG |
| SspE_R133A_-2 | TGTAGTCAATGCTTGTTGACCATCTATTATAGT |
| **Construction of pWHU6004 and pWHU6007** | |
| SspE_R724A_-1 | GGTCAACAAGCATTGACTACATTGCTATTAGTG |
| SspE_R724A_-2 | TGTAGTCAATGCTTGTTGACCATCTATTATAGT |
| **5'-C_PS_CA-3′/5′-CCA-3′-containing DNA** | |
| 5′-C_PS_CA-3′ | 5′-TGTGGCAAAGAATCTATTGC_PS_CAGAAGCCAGT  TTCGATACT-3’  3’-ACACCGTTTCTTAGATAACGGTCTTCGGTCGGTC  AAAGCTATGA-5’ |
|  |  |
| 5′-CCA-3′ | 5′-TGTGGCAAAGAATCTATTGCCAGAAGCCAGT  TTCGATACT-3’  3’-ACACCGTTTCTTAGATAACGGTCTTCGGTCGGTC  AAAGCTATGA-5’ |
