# Supplementary material for: SspE-mediated immune defense: GTP hydrolysis as an allosteric switch coupling phosphorothioate recognition to DNA cleavage
Source: mBio. 2026 May 12;17(6):e00359-26. doi: 10.1128/mbio.00359-26 (PMC13251355; doi:10.1128/mbio.00359-26)
Supplement: Table S3 — Cryo-EM data collection, refinement, and validation statistics. [file mbio.00359-26-s0006.docx]

| **Sample** | **WT** | **R133A** | |
| --- | --- | --- | --- |
| **Data collection and processing** | | | |
| EM equipment | Titan Krios (Thermo Fisher Scientific) | | |
| Voltage (kV) | 300 | | |
| Detector | Gatan K3 | | |
| Pixel size (Å) | 0.85 | | 0.85 |
| Defocus range (µm) | -0.8~-2.7 | -0.7~-2.6 | |
| Magnification | 105,000 × | 105,000 × | |
| Frames | 32 | 32 | |
| Total dose (*e^−^* /Å^2^) | 48 | 45 | |
| Number of collected micrographs | 1,800 | 2,030 | |
| Number of used particles | 216,505 | 179,737 | |
| Map Resolution (Å) | 3.28 | 2.81 | |
| Symmetry | C1 | C1 | |
| **Refinement** | | | |
| Initial model | Alpha Fold | Alpha Fold | |
| Model Resolution (Å) | 3.3 | 2.9 | |
| Model composition | | | |
| Chains | 4 | 4 | |
| Non-hydrogen | 13,029 | 17,004 | |
| Residues | Protein: 1,583 | Protein: 2,077 | |
| B factors (Å^2^) | | | |
| Protein | 66.29 | 28.29 | |
| Ligand | -- | 14.69 | |
| Water | -- | -- | |
| R.m.s. deviations | | | |
| Bonds (RMSD) | 0.002 | 0.003 | |
| Bonds length (Å) | 0.487 | 0.535 | |
| Validation | | | |
| MolProbity | 1.52 | 1.53 | |
| Clashscore | 6.36 | 4.14 | |
| Rotamer outliers (%) | 0.28 | 2.78 | |
| Ramachandran plot statistics (%) | | | |
| Preferred | 97.30 | 98.26 | |
| Allowed | 2.97 | 1.74 | |
| Outlier | 0.00 | 0.00 | |

**Table S3. Cryo-EM data collection, refinement and validation statistics.**
